# Survival After Diagnosis of Esophageal Squamous Cell Carcinoma in Malawi

**DOI:** 10.1200/GO.23.00173

**Published:** 2023-11-09

**Authors:** Bongani Kaimila, Yingxi Chen, Gift Mulima, Chifundo Kajombo, Ande Salima, Yukiko Yano, Satish Gopal, Sanford M. Dawsey, Christian C. Abnet

**Affiliations:** ^1^ UNC Project, Lilongwe, Malawi; ^2^ Division of Cancer Epidemiology and Genetics, National Cancer Institute, National Institutes of Health, Rockville, MD; ^3^ Kamuzu Central Hospital, Lilongwe, Malawi; ^4^ Center for Global Health, National Cancer Institute, National Institutes of Health, Rockville, MD; ^†^ Deceased

## Abstract

**PURPOSE:**

Esophageal cancer (EC) is the second most common cancer in Malawi, with esophageal squamous cell carcinoma (ESCC) representing >90% of all ECs. Despite significant morbidity and mortality, little is known about disease outcomes. In this study, we assess survival after ESCC diagnosis in Malawi.

**METHODS:**

We report on ESCC cases enrolled in a case-control study at Kamuzu Central Hospital in Lilongwe from August 2017 to April 2020. Suspected cases completed a questionnaire interview; provided blood, urine, and saliva specimens; and underwent a tumor biopsy for histologic confirmation. Cases were followed up by phone biweekly from enrollment to the study end date (December 31, 2020), date of death, or loss to follow-up. Survival was assessed using Kaplan-Meier analysis with the log-rank test. We also examined associations between treatment and ESCC mortality using Cox regression models.

**RESULTS:**

There were 300 patients with ESCC enrolled in this study, of whom 290 (97%) had known vital status at the end of follow-up and 10 (3%) were lost to follow-up. Among the 290 patients, 282 (97%) died during follow-up. The median age at enrollment was 55 years (IQR, 48-66), and the median time to death was 106 days (95% CI, 92 to 127). The 1-year, 2-year, and 3-year survival rates were 11% (95% CI, 8 to 15), 3% (95% CI, 1 to 6), and 0.9% (95% CI, 0.8 to 4), respectively. Palliative chemotherapy significantly improved the overall survival of patients with ESCC (*P*_log-rank_ = .038) and was significantly associated with reduced mortality (adjusted hazard ratio, 0.71 [95% CI, 0.51 to 0.99]). No significant association was observed between tobacco use, alcohol consumption, or HIV status and mortality.

**CONCLUSION:**

Survival after diagnosis of ESCC was poor in Malawi. Although palliative chemotherapy was associated with improved survival, prevention and earlier detection remain key priorities to improve ESCC mortality at a population level.

## Teaser

The survival rate of ESCC was very low in Malawi. Screening and early detection are critical to reduce mortality.

## INTRODUCTION

Esophageal cancer (EC) is the sixth leading cause of cancer death globally.^1^ A recent meta-analysis of EC in Africa reported that within sub-Saharan Africa, Malawi has the highest incidence rate for both men (30.3 cases per year per 100,000) and women (19.4 cases per year per 100,000) between 2009 and 2013.^2^ Classified as esophageal squamous cell carcinoma (ESCC) or adenocarcinoma, EC is the second most common cancer and the most common non-HIV–related cancer in Malawi.^3-5^ Despite the high burden, there is very little literature on treatment and survival of patients with EC in Malawi or elsewhere in Africa.^6^

**CONTEXT**

**Key Objective**
What is the survival of the patients diagnosed with esophageal squamous cell carcinoma (ESCC) in Malawi?
**Knowledge Generated**
In our study, the overall survival rate for ESCC in Malawi was very low. This poor survival was most likely due to the advanced stage of disease at presentation, with 82% of patients presenting with complete obstruction. Receipt of palliative chemotherapy was associated with modestly longer survival, although long-term survival of ESCC remained very poor.
**Relevance**
Improving treatment access remains an important priority to improve outcomes for patients with ESCC in Malawi. At the population level, effective and implementable screening and early detection methods will be critical to reduce ESCC morbidity and mortality. Additionally, continued etiologic studies are essential to identify potential opportunities for prevention.

Worldwide, surgery has historically been considered the primary mode of curative treatment for localized tumors.^7^ For advanced disease, chemoradiation has historically been the main palliative treatment. In settings where chemoradiation is not available, self-expanding esophageal metal stents (SEMS) are used for palliation.^7-9^ More recently, immunotherapy and molecularly targeted agents have shown promise.^9^ Despite advances in care, outcomes for patients with ESCC remain poor, although there are marked global variations correlated with the level of economic development. For example, the median survival of ESCC is 12 months in Taiwan compared with 4 months in Ethiopia.^10,11^

In Malawi, endoscopy services are limited to tertiary public hospitals in Blantyre, Zomba, and Lilongwe. Diagnostic endoscopy services are not available in any of the public district hospitals or health centers,^12^ and screening endoscopy services are not available anywhere in the country. Barium meal imaging is considered the first diagnostic step for ESCC in most district hospitals, followed by referral to tertiary public hospitals for diagnostic endoscopy. Very few patients are diagnosed at a stage when curative therapy can be considered. After diagnosis, most patients are considered for SEMS or chemotherapy as palliative options, which are administered at two tertiary public hospitals, Queen Elizabeth Central Hospital (QECH) in Blantyre and Kamuzu Central Hospital (KCH) in Lilongwe. Surgical esophagectomy is currently available only at QECH, and radiotherapy is not currently available in Malawi.

This study describes the outcomes of patients diagnosed with ESCC at KCH in Lilongwe, Malawi, from 2017 to 2020. We also examine associations of treatment and patient characteristics with ESCC mortality.

## METHODS

### Study Setting and Population

From August 1, 2017 to April 4, 2020, patients age 18 years or older with ESCC at KCH and St Gabriel Hospital (SGH) in Lilongwe, Malawi, were enrolled into a case-control study^13^ and prospectively followed. KCH is a referral teaching hospital in the capital, Lilongwe, with endoscopy services and Malawi's National Cancer Centre. KCH provides palliative services including SEMS and chemotherapy to patients with ESCC. SGH is a secondary health care facility 45 km from KCH and offers endoscopy and SEMS only, referring patients who require other services to KCH.

### Study Procedures

We enrolled ESCC cases from participants with dysphagia attending endoscopy at KCH or SGH. Evidence of an esophageal tumor on endoscopy was required to be enrolled in this study. ESCC diagnosis was confirmed by histopathology of tumor biopsies obtained during endoscopy. Detailed methods are described elsewhere.^13^ To summarize, all adults age 18 years or older presenting to the KCH and SGH endoscopy units with symptoms suggestive of ESCC were screened for this study. Patients were enrolled before endoscopy to facilitate consent for research specimen collections during the procedure. Owing to the scope of our project, we only included patients with ESCC in this study, and all other histological types were excluded. In our hospital, most patients had squamous cell carcinomas, which is consistent with published data.^14^ There were no pediatric patients, and all eligible patients consented to participate. Furthermore, on enrollment, there were no patients eligible for esophagectomy; therefore, no participants were referred to another center for treatment. Enrolled participants had demographic information recorded in an electronic database, and a paper-based physical location map was also recorded. We collected data on demographic characteristics and relevant health behaviors using a computerized interviewer-administered questionnaire to assess age, sex, tobacco smoking, alcohol consumption, HIV status, and dysphagia score. Smoking was defined as the total lifetime use of tobacco products for ≥6 months. Alcohol consumption was defined as the total lifetime consumption of alcoholic beverages for ≥6 months. HIV status was obtained from patient health records. The dysphagia score was used to estimate disease severity.^15^ This score is based on the symptoms caused by tumoral stricture. A score of 0 indicates able to eat normal diet; 1, able to swallow some solid food; 2, able to swallow only semisolid food and liquids; 3, able to swallow only liquids; and 4, complete dysphagia, meaning unable to swallow liquids or saliva.^15^ Clinical staging was not performed because of inconsistent availability of computed tomography.

After enrollment, phone follow-up for vital status and receipt of treatment were conducted every other week. If a participant was unreachable by phone on four consecutive attempts, a physical follow-up visit was attempted. Participants not reachable by physical follow-up were assigned as lost to follow-up on the last date of contact. Treatment was provided as per local standards of care after provider-patient consultation. SEMS and/or chemotherapy were typically offered first when feasible, and pain management alone was typically offered when these were not feasible. Treatment was recorded as SEMS, chemotherapy, pain management only, others, or unknown. Depending on availability, we used two palliative chemotherapy regimens: the carboplatin and paclitaxel combination and the fluorouracil and cisplatin combination. Carboplatin can be interchanged with cisplatin, and paclitaxel can be replaced with docetaxel if necessary.

### Statistical Analyses

We used simple descriptive statistics to characterize the cohort and Kaplan-Meier methods to estimate survival. We used log-rank tests to compare survival on the basis of treatment received. Follow-up was calculated from the date of recruitment until death, loss to follow-up, or administrative censoring on December 31, 2020, whichever occurred first. To further estimate associations of patient characteristics and treatment with mortality, we used univariate and multivariate Cox proportional hazards regression to calculate hazard ratios (HRs) and 95% CIs. We conducted univariate Cox regression to estimate crude HRs, separately for age, sex, smoking, alcohol consumption, HIV status, dysphagia score, and treatment modality. Significant variables (defined as *P* < .25 based on the stepwise regression method) from univariate analysis were included in the final multivariate Cox regression model. We conducted the Schoenfeld residuals test to test the proportionality assumption of the Cox regression models (*P* > .05 indicates no violation of the proportionality assumption). All analyses were conducted in Stata 14 (College Station, TX).

### Ethics

All patients included in this study provided written informed consent. The study was approved by the Malawi National Health Sciences Research Committee and the University of North Carolina Biomedical Sciences Institutional Review Board (Protocol # 16/07/1633 LCCC 1608). NCI staff participated without access to personally identifying information or direct patient contact.

### Funding

The study was sponsored through the Malawi Cancer Consortium and in part by the Intramural Research Program of the National Cancer Institute, National Institutes of Health.

## RESULTS

Between 2017 and 2020, we enrolled 300 patients with suspected or confirmed ESCC, of whom 290 (97%) had known vital status at the end of follow-up and 10 (3%) were loss to follow-up after the date of enrollment. The main reason for loss to follow-up was relocation of patients to new homes. Among the 300 patients at enrollment, the median age was 55 years (IQR, 48-66 years), 169 (58%) were male, 31 (10%) were HIV positive, 212 (75%) were married, and 99 (35%) had a secondary or tertiary education (Table 1). A total of 35 patients (12%) were younger than 40 years. Histological confirmation of ESCC was available for 258 (86%) patients while 42 (14%) patients had an inadequate biopsy but endoscopic assessment consistent with ESCC. A dysphagia score of ≥3 (able to swallow liquids only) was present in 282 (97%) patients.

**TABLE 1:**
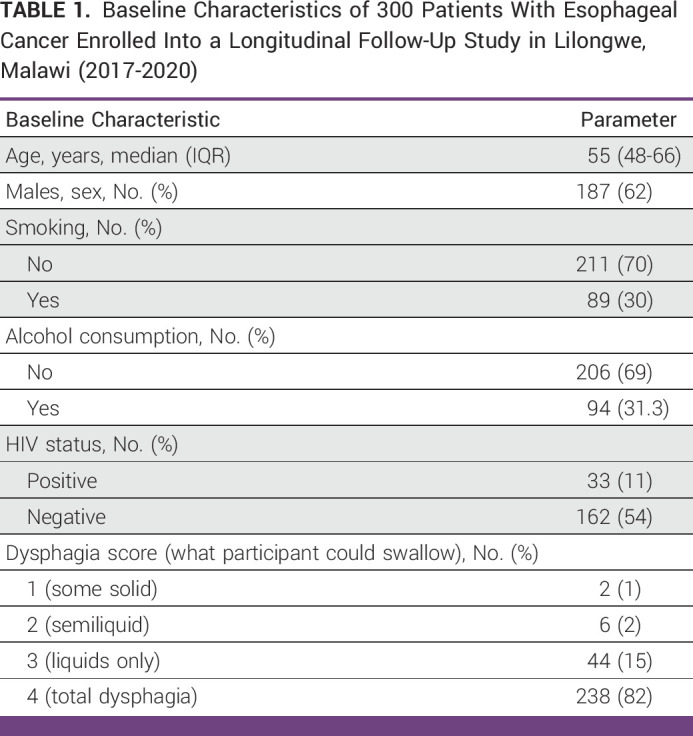
Baseline Characteristics of 300 Patients With Esophageal Cancer Enrolled Into a Longitudinal Follow-Up Study in Lilongwe, Malawi (2017-2020)

**FIG 1:**
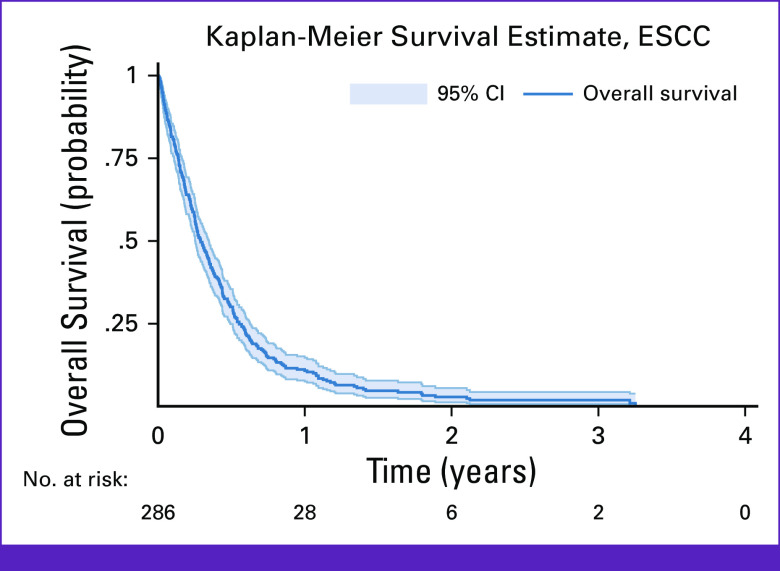
Kaplan-Meier survival curve of the overall survival pattern among patients with ESCC enrolled in a longitudinal follow-up study in Lilongwe, Malawi (2017-2020). ESCC, esophageal squamous cell carcinoma.

At the end of follow-up, 282 (97%) patients had died and eight (3%) were alive. The median follow-up time was 105 days (IQR, 49-202 days), and the median time to death was 99 days (IQR, 46-190 days). Overall, the 1-year, 2-year, and 3-year survival estimates were 11% (95% CI, 8 to 15), 3% (95% CI, 1 to 6), and 0.9% (95% CI, 0.8 to 4), respectively (Fig 1). A total of 76 (26%) patients received palliative SEMS alone, 36 (12%) patients received palliative chemotherapy alone, and eight (3%) patients received both SEMS and chemotherapy. For the 206 patients who did not receive palliative SEMS, 69 (34%) patients did not consent to SEMS placement, and 51 (25%) patients had a tumor which was unamenable to SEMS placement.

**FIG 2:**
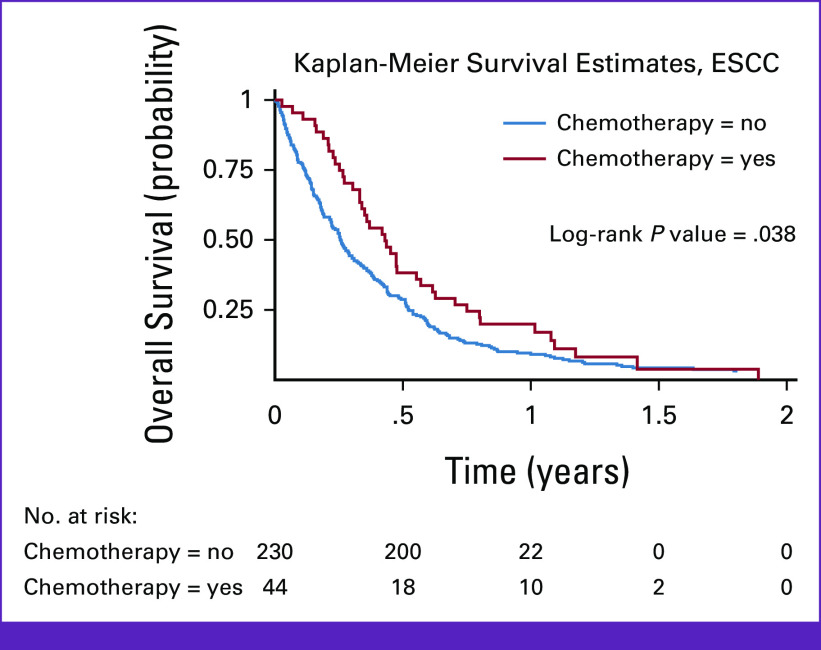
Kaplan-Meier survival curve stratified by palliative chemotherapy treatment, among patients with ESCC enrolled in a longitudinal follow-up study in Lilongwe, Malawi (2017-2020). ESCC, esophageal squamous cell carcinoma.

In Kaplan-Meier analysis, palliative chemotherapy was significantly associated with improved overall survival (OS; *P*_log-rank_ = .038; Fig 2). We did not observe statistically significant differences in age (*P* = .31), sex (*P* = .17), place of origin (*P* = .65), or socioeconomic status (*P* = .08) between patients who received palliative chemotherapy and those who did not. Patients who received palliative chemotherapy had a lower median travel time of 1 hour (range, 0.5-4 hours) from their home to KCH compared with patients who did not receive palliative chemotherapy, who had a median travel time of 2 hours (range, 0.15-11 hours).

In univariate Cox regression, palliative chemotherapy was significantly associated with reduced ESCC mortality (HR, 0.70; 95% CI, 0.50 to 0.98; Table 2). HIV status (*P* = .40), smoking (*P* = .25), and alcohol consumption (*P* = .88) were not associated with ESCC mortality in univariate analysis and were not included in the final multivariate Cox model (Table 2). Age, sex, and dysphagia score were included in the final multivariate Cox model. After adjustment, age (*P* = .11), sex (*P* = .14), and dysphagia score (*P* = .17) were not significantly associated with ESCC mortality (Table 2). Compared with patients who received pain control only, there was no statistically significant difference in mortality across specific palliative treatment modalities received, although any receipt of chemotherapy overall across treatment plans was associated with reduced mortality (adjusted hazard ratio [aHR], 0.71; 95% CI, 0.51 to 0.99; Table 2). The Schoenfeld residuals test indicated no significant violation of the proportionality assumption of the final model (*P* = .28).

**TABLE 2:**
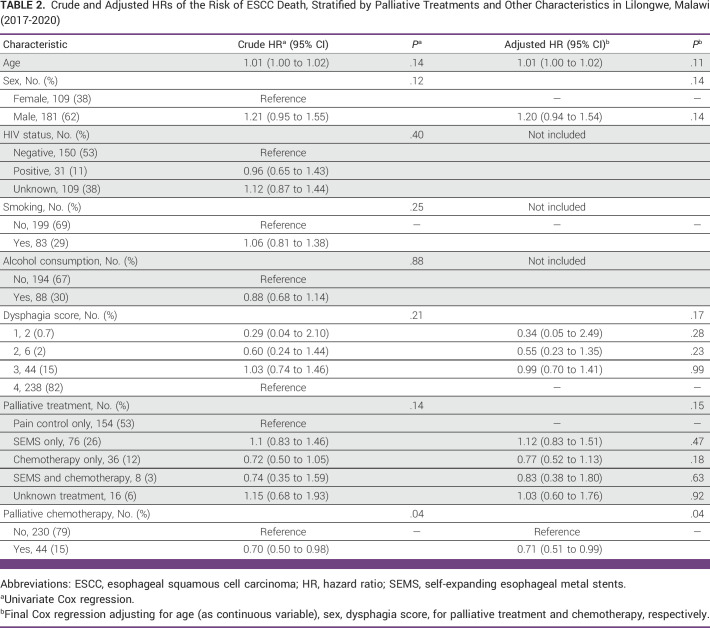
Crude and Adjusted HRs of the Risk of ESCC Death, Stratified by Palliative Treatments and Other Characteristics in Lilongwe, Malawi (2017-2020)

## DISCUSSION

In our study, the OS rate for ESCC was very low in Malawi, comparable with other African settings.^11^ The poor survival was likely due to advanced disease stage at presentation. Although formal staging was not performed, 82% of patients presented with complete obstruction. Another probable contributor to poor survival was limited treatment access. Treatment in Malawi is largely palliative with more definitive treatment options such as esophagectomy or radiotherapy being unavailable to most patients.

Heavy alcohol intake has previously been associated with poor ESCC outcomes.^16-18^ The limited number of heavy drinkers in this likely explains our observed lack of association between alcohol and ESCC mortality, given that most patients were either never drinkers or light drinkers. Prediagnosis smoking has also been associated with worse outcomes of ESCC.^16,17^ We did not observe differential mortality among smokers and nonsmokers. This may have been due to advanced disease and poor performance status for most patients on enrollment in our cohort, which resulted in early mortality and masked potential effects of smoking on survival. Furthermore, our relatively small sample size may have limited our statistical power to detect significant effects.

Receipt of palliative chemotherapy, however, was associated with modestly longer survival in our study, although this is based on a relatively small number of patients (approximately 15% of the cohort) who received chemotherapy and who may have differed from other patients in ways that were not directly measured. Combination palliative chemotherapy has been reported to improve OS for patients with advanced EC.^19^ However, long-term survival of ESCC remained very poor in our study, and the application of chemotherapy for advanced ESCC in Africa deserves additional study to comprehensively capture costs and benefits at the individual and health system levels, to appropriately inform regional policy and practice.

In our study, treatment with SEMS alone was not associated with improved OS for patients with ESCC, which is consistent with published data.^20^ SEMS placement has been reported to improve survival when administered in combination with other therapies.^21-23^ We noted that patients in our cohort who received SEMS in combination with chemotherapy did experience lower ESCC mortality compared with pain control only, but this difference was not statistically significant. This may have been due to the small number of patients who received this combined modality treatment. Furthermore, quality of life (QOL) was not assessed in this study, and therefore, differences in QOL across treatment options were not evaluated, which is a notable omission, given the largely palliative treatment approaches available for patients with ESCC in Malawi and elsewhere in Africa. Nevertheless, improvements in QOL have been reported for SEMS recipients,^24^ and more research focused on QOL for patients with ESCC is needed in Africa to inform regional policy and practice along with shared decision making between patients and providers.

In conclusion, survival of patients with ESCC in our study was poor. Advanced disease at presentation was likely the primary contributor to poor survival. Limited treatment options, especially the lack of esophagectomy and radiotherapy, likely also contributed to poor outcomes. Improving treatment access remains an important priority to improve outcomes for individual patients. At a population level, effective and implementable screening and early detection methods will be critical to reduce ESCC morbidity and mortality in Malawi, along with continued etiologic studies to identify potential opportunities for prevention.
